# Salsa Rhythms and Soul Connections

**DOI:** 10.1177/10778004231176094

**Published:** 2023-06-03

**Authors:** Rebecca J. Lloyd, Stephen J. Smith

**Affiliations:** 1 University of Ottawa, Ontario, Canada; 2 Simon Fraser University, Burnaby, British Columbia, Canada

**Keywords:** relational reflexivity, flow, rhythm, salsa dance

## Abstract

The rhythmic interplay of accent, tempo, and musical mood is expressed in the bodily postures, gestures, and expressions of attuned responsiveness in *Salsa Dura*, a genre of salsa music from the 1970s featuring improvisational dance solos. These dancers embrace the feelings and flows of soloing musicians *going off* and breaking free from any predictable form and structure. We inquire into how world-class salsa dancers and educators feel themselves moved by such intricate rhythms to experience soul connections. Video recordings and interviews yield insight into the call and response dynamics of this essentially tactful practice of alterity.

It is out of relational process that what we call the person takes form. Whether we consider the essence of the person as a soul, a conscious decision-maker or a brain in action depends on the tradition of meaning-making in which we participate. . . Bodies both enable and limit our movements but all they do in terms of meaningful action emerges from relational process. (Gergen, 2021, p. 22)

“Celine, not the singer” is how my new 72-year-old French Canadian friend introduces herself to me on the boardwalk. She asks, “Est ce que tu connais le cha-cha? I nod YES with a smile. “Va a le DJ et demande un cha-cha.” I comply. Within moments, my weight transfers pick up the “step-step 1,2,3” rhythm. And Celine? Well, let’s just say she moves happily to the beat of her own drum. Like children engaged in parallel play, our actions and interactions are not coordinated, yet we share something very special—a moment of joyful release to Latin music on a glorious sunny afternoon. A day later, we meet poolside to dance again and are joined by another woman who is new to dance. The teacher at the resort rhythmically breaks down the 1, 2, 3 tap of a bachata song in exaggerated side-to-side steps. The woman follows with some trepidation and, by the end of the song, exclaims, “I am too White for this!” The Cuban teacher responds by turning to me, a Latin dancer of White British descent, with open arms as a salsa song begins to play. I move in closer to accept his embrace. An interplay of lead–follow movements ensue—back steps, turns, and side-to-side sways—with wordless grace. (Lloyd, travel journal, 2023)

Kenneth Gergen (2021) infers in the epigram with which we open this article that, through the gestural dance of interactive bodies, we may develop a deep understanding of one another. Yet, Gergen, as well as other academics who turn to the metaphor of dance to address relational matters, stops short of the nuanced ways dancers engage in motile conversation. Often categorized under the generic label of *nonverbal* communication, it is as if something magical happens when people start dancing together. Little consideration is generally given, outside arts-based and somatic inquiry, to the expressive bodily contours of rhythmic connection (Cancienne & Snowber, 2003; Sheets-Johnstone, 2011; Stern, 2010) and the kinesthetically and energetically felt dynamics of dancing across bodily cast and complexioned differences (Bergonzoni, 2022; Snowber, 2011).

Moving to music holds a number of relational possibilities. One might be open and free like septuagenarian Celine; others may rigidly hold onto beliefs that they are not dancers (Leonard et al., 2022) or that movements of the hips are reserved for people of particular cultures (Schupp, 2020). Even if one is comfortable enough to accept a partnered dance, mere physical connection is not enough to ensure it will go well. A lead can be too rough, a follow too tense. One or both may not coordinate with the music. To find what Gergen (2021) describes as “a mutually agreeable way to dance together” (p. 28) across gendered and cultural differences (McMains, 2018), the lead and follow must cultivate a “somatic movement literacy” (Buono, 2022, p. 317) with rhythmic sensitivity to the timing, force, direction, and amplitude of their partnered movements.

Awareness of such rhythmic sensitivities in the presence of others, and the “relational affects” (Stern, 1993, p. 211) each accented moment carries, is worthy of further inquiry if we are to understand how dance can contribute positively to what Gergen (2021) describes in the title of his book as a *world on edge*. Can we countenance the affectivities of inherently relational existence amid differences that matter at this present time of such divisiveness? This is the question we take up indirectly, which is to say kinetically and kinesthetically, in an exploration of the manner in which rhythmicity moves dancers in moments of life-altering self-transcendence to where the “extended self” (Stern, 1993, p. 213) experiences “higher levels of activated joy” through rhythmic inter-action (p. 209).

## Reflexive Motional Inquiry

We come to this reflexively relational, rhythmical dance inquiry through various partnered practices including salsa which Rebecca, the first author, has pursued competitively and written about (i.e., Lloyd, 2015a, 2015b, 2017, 2021) and equestrian arts in which Stephen, the second author, has done likewise (Smith, 2014, 2015a, 2015b, 2018, 2019; Smith & LaRochelle, 2019). Engaging in such dynamically relational practices, we appreciate the postural, positional, gestural, and expressive cultivation of movement joy beyond what we can experience on our own. We are thus moved not only to experience partnered practices for the pleasures individually derived but also to further kinetic, aesthetic, kinesthetic, and energetic ways of moving joyfully with others. Over the past 5 years, within our Social Sciences and Humanities Research Council–funded multiphase InterActive for Life (IA4L) project, we have observed, interviewed, and made video documentaries of expert practitioners of partnered movement practices ranging from Push-Hands Tai Chi, to AcroYoga, Salsa Dance, and Equestrian Arts (Phase 1); mobilized our findings to enhance pedagogical practices of Teacher Education students through the co-creation of generalized partnered activities and resources (Phase 2); and responded to emerging and seasoned educators with vested interest in prioritizing motile communication and relational connectedness in their pedagogical practices (Phase 3); Lloyd, 2021; Lloyd & Smith, 2021, 2022a, 2022b; Nyetap et al., 2020; Smith & Lloyd, 2022).

Attention has been given, for instance, to the ways we communicate readiness to give and receive information through relational postural alignment, muscular tone and tension, and the various inclinations and positions that convey meaning (Lloyd & Smith, 2022a, 2022b). But we have not necessarily focused on the rhythmical ways in which these postures and positions are enlivened. It is not that we want to hold back on entertaining the cadenced feelings and rhythmic flows that make partnered practices so joyful. As much as we are drawn to motions of vital expression, such as the shimmying of shoulder blades in Salsa, we have been mindful of the readiness of teacher candidates who participated in our IA4L project to embrace these affective and synergistic movement qualities. Despite backgrounds in sports, fitness, and recreational activities, it is the first-time beginning teachers have been asked to consider movement primarily as a language of connection. When learning to play basketball in school, for example, student focus is directed to the ball and where it eventually needs to go to score a point. No instructor ever took the ball away and invited students to explore the dynamic dance of postural and positional weight transfers in offensive and defensive play. As somatically oriented educators such as Sondra Fraleigh (2015) recommend, we have s-l-o-w-e-d down to help these student teachers feel the profound *vitality affects* (Sheets-Johnstone, 2011) of something as simple as a forward lean when facing a partner (Lloyd & Smith, 2022a) in a variety of lead–follow games that they have co-created (i.e., Leaning in Mirror Walk; Nyetap et al., 2020; Lloyd & Smith, 2022b; Smith & Lloyd, 2022).

As we now conclude the final phase of this relational study, we want to inquire further into the rhythmic dynamics of motional communication. Attending to the relational postures and positions is not enough. We take seriously, through a dance exemplar of motional connectivity, rhythmicity as an essential communicative dimension. Our intention is to describe how rhythmicity is the dimension of tangible affectivity wherein self and other do not stand apart distinctly and differently but meet, match, and merge with one another in motional responsiveness. It is to free up a critically reflexive stance that can hold itself too rigidly amid matters of differently inscribed and diversely complexioned bodies and come more motilely and rhythmically to where fluid connectivity can be felt as a “pathos-with” with others (Henry, 2008, pp. 101–134).

## Relational Consciousness

The most obvious relational connection in Salsa dance is between the *lead* and the *follow* (Harman, 2019; Lloyd, 2021; McMains, 2018). Subtle shifts of weight, bodily positioning, and movements of the limbs, palms, and even fingers communicate intention affectively and effectively in the recognizable salsa rhythm of 1, 2, 3 5, 6, 7.^1^ Less obvious, but of equal importance, are the connections felt between the dancers and those playing the salsa music. Despite the possibility of being moved beyond the 1, 2, 3 5, 6, 7 measures and by the lyrics, melodies, or accents communicated in each song, novice *lead* and *follow* dancers will tend to be so preoccupied learning a few basic patterns that the basic rhythm is all they hear.

The more one commits to the practice of salsa dance, the more prominent the lead–follow dynamics in relation to the rhythmicity of the music being played. Metronomic measures of the predictable 1, 2, 3 5, 6, 7 salsa rhythm give way to a tactilely felt interplay. Musicality as specifically rhythmicity is an inter-animation of dancers and musicians. It becomes a conversation between those playing specific instruments or singing lyrics or making scatting sounds and those on the dance floor. For example,

A Cuban seven-piece band plays for couples improvising on the dance floor. Midway into their third set, a group of us dancers gather to form a semi-circle around their stage. We shimmy, sway and clap in time to “Ba-Da DaDaDaa, Ba-Ba DaDaDaDaa,” a rhythm playfully passing back and forth, like an invisible ball, between the lead singer and trombonist.

Birthday girl Samantha—professional dancer, multi-world salsa champion, international judge and coach— gravitates toward this rhythmic interplay. She joins our semi-circle with a wide-eyed smile and picks up the rhythm with her feet and hips. The lead singer notices her presence not only in his welcoming smile and extended arm accentuating the accents he is producing with his voice, but also in the lyrics rippling from his lips: “Felicidad Hey Sa-man-tha. . . . Ba-Da DaDaDa Ba-Da DaDaDa; Felicidad Hey Sa-man-tha. . . . Ba-Da DaDaDa Ba-Da DaDaDa.” Samantha responds by stepping out of the semi-circle toward the singer and increases the intensity and amplitude of her serpentine sway. The singer steps closer to Samantha and the exchange between dancer and singer builds in energy and deepens in felt sensation. A blurring of call and response is felt by all who are in the energetic wake of this exchange. (Lloyd, dance journal, 2022)

**Figure 1.**
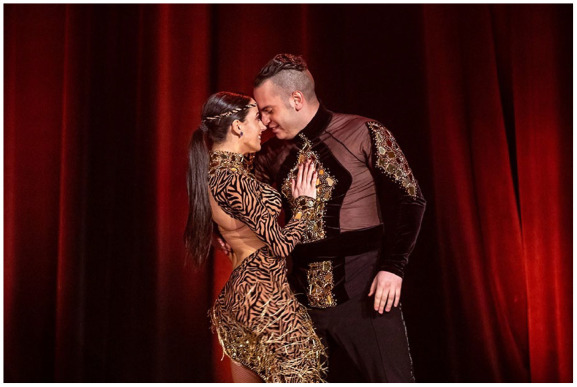
Samantha Scali and Adriano Ieropoli, 5-Time World Salsa Champions, Owners of Novaera Productions.

What washes over those who experience this moment of music–movement responsivity is representative of the historic roots of salsa dance. Five-time world champions Adriano Ieropoli and Samantha Scali (Figure 1), known for their interpretations of intricate, fast-paced salsa rhythms—who accepted an ethics-approved invitation to participate in our IA4L project and be featured in a mini documentary (see https://vimeo.com/786445887/1ab9bc17f7)—explain the call and response dynamics. Ieropoli says,

Salsa stems from African dances where the response pattern is actually inversed. The dancer moves and the drums respond to the dancer, which is completely the reverse of how we dance to a song that is prerecorded. There are African dances where the dancer actually conducts the music. (Personal communication, March 5, 2022)

Scali adds, “That is the background to what we dance. It is a fusion over centuries” (personal communication, March 5, 2022)

Knowledge of salsa music is important for appreciating its origin in the call and response dynamic that has informed the commercialized version of Mambo we hear today, a musical genre and dance that dates back to the 1930s. Made famous in the 1950s by singers such as Tito Puente, the King of Latin jazz, the Afro-Cuban and Caribbean sounds, with roots in the *candombe* form that draws upon West African rhythms, morphed into the generic term *Salsa* in the 1970s to 1990s, yet its history cannot be forgotten. As Kabir (2020) acknowledges, salsa and other “African-heritage partner dances” enact “the traumatic processes of enslavement, colonialism, and extractive capitalism” while at the same time they may be understood as “embodied and mobile archives: kinetoscapes of newness and expressivity that arise in response and resistance to cultural deracination” (p. 2). Within such call and response dynamics, we sense the soul, not only of the musicians but of the dancers who resisted the colonizers’ dehumanizing perspective that Africans they encountered did not have souls (Bordas, 2012; Du Bois & Marable, 2015). For those wishing to find ways to move through such horrific tensions, listening and moving to salsa music has the potential for “bringing people together in rhythmic affinity” in spite of racial dissimilarities and as “a new way of thinking about politics and culture” (Kabir, 2020, p. 9). Joyous rhythms defy the oppressive hold of the plantation, the camp, the prison, and the ghetto (Kabir, 2020, p. 7). They permit nowadays the mingling and merging of those whose gender, ethnicity, age, and even ability might well keep them apart.

## Shining in *Salsa Dura*

One can see when the call of distinct instruments and voices is being heard by dancers because their movements exude a very different quality. The manner in which a cue is given or received changes in relation to the dynamic accents emphasized within the dance and the song. Rote movements to salsa rhythms transform from that which are predictable and reproducible to where dancers move *with* discernible variations in expressive quality and vitality. When this *conversation* happens, a language of feeling becomes visible and palpable in the movements themselves.

When the music is particularly good, when “all the ingredients are cookin’ just right” and it exudes a certain amount of heat—a quality of the sauce Izzy Sanabria ascribed to the dance for marketing purposes (Renta, 2014, p. 119)—salsa dancers release from their partners and respond to the call of the music. They dance freely in side-by-side improvisational movements of intense musical connection. These moments are known as *shines* and are more than what dancers do just with the music. They, the dancers, merge with the salsa rhythms and invite certain instruments to speak directly to their bodily motions without, as other somatic movement educators have indicated (Williamson & Sellers-Young, 2020), letting their thinking minds get in the way.

Some songs are softer and fall within the *Salsa Romantica* genre. *Salsa Dura*, on the contrary, is a style that is more likely to inspire a salsa dancer to shine as it is composed of a series of instrumental solos that, according to Samantha Scali and Adriano Ieropoli, is their favorite kind of salsa music for dancing:

What I love about *Salsa Dura* is that it has this rough, really authentic and raspy sound that we don’t hear nowadays. The songs will often pick up in tempo. They’ll start slower and [get] crazy fast by the end of the song. There are variations of rhythms and what I really love is that there are a lot of solos. (Scali, personal communication, March 5, 2022)

Ieropoli adds,

We call them *Descargars* which is like saying a solo for a specific instrument. . .You will hear the piano solo, the timbale solo, the conga solo. They will literally play one after another. (Personal communication, March 5, 2022)

Scali is asked what it is about these solos that excites her so much:

I feel like I’m connecting to what the musician was feeling in the moment he was recording this solo. You can tell if the musicians go to another place because they are not following the basic rhythms. They’re not necessarily playing the same riffs that they were playing in other sections of the song. When musicians have their souls in it, they can really “go off,” as we say, and they really mix it up by adding so many different sounds and variations to the rhythms. It makes it very exciting . . . and you want to just stay up there in terms of energetic connection with the song. (Personal communication, March 5, 2022)

These soloing moments are both the most challenging and appealing ones of *Salsa Dura*.

## A Time to Shine

How do dancers learn to shine? Do we begin by simply moving to music or learning the basic steps with the intention of one day breaking free from rhythmic constraints? Might we hear more than one blurred sound and begin to pick up on the dynamic conversations between instruments in our movements? What level of openness is required for music to be given the designated role of lead where mood, feelings, and exchanges of energy may flow? Sadly, in Western culture, such a release and giving over of control to be moved by music is mostly experienced artificially in the use of drugs and alcohol as there are so many imposed barriers to improvisational participation in dance (Leonard et al., 2022).

Beginning dance education early in schools could be one way of cultivating a rhythmic, somatic literacy (i.e., Buono, 2022) as children, as Scali suggests, exude an openness to move. She says tellingly,

You can see it when you put on music and children dance. They just move. Often they haven’t had lessons, they don’t know what they’re doing, but they just move to the rhythm. (Scali, personal communication, March 5, 2022)

To which Ieropoli adds,

And they allow themselves to move also without judgment or without thinking so much and that’s why it’s so natural. You see how much fun they’re having which is what we don’t want to lose as we age. But a lot of time we create blockages or barriers because we think we’re not good enough. Those barriers are really what’s blocking you from allowing the soul to really come out and for you to discover who you are as a dancer, or even just as a person. (Personal communication, March 5, 2022)

What Ieropoli is describing, this genuine connection to salsa rhythm, without blockages or barriers, is exemplified in a YouTube video of him dancing a shine solo in the men’s division of the 2019 World Salsa Summit (see https://www.youtube.com/watch?v=tDNnOavNk-w). Here we see Ieropoli walk onto the world competition stage. He stands tall, opens his arms to address the judges and audience with a firm nod, and, with his head held high, walks down the stairs to assume his place on the dance floor. He traverses the space in variations of salsa rhythm where his feet cross, knees bend, shoulders shimmy, and fingers point to punctuate accents. The speed and clarity of his seemingly effortless footwork continue until there is a moment of suspension. The trumpet wails, the singer’s voice expands, and, between 1:46 and 1:52 s, Ieropoli glides softly from side to side and then lifts his arms and closes his eyes. While nothing overly spectacular is happening in terms of a trick such as a jump, kick, or series of turns, the audience raise their voices in a loud cheer. What is happening that is creating such a stir? The dancer is simply lifting his arms and closing his eyes. To get to this level of soul connection, where intonations such as the sustained wail of a trumpet becoming visible in the quality of one’s movements, requires commitment to a regular practice. In terms of the balance between training technique and opening oneself up in moments of musical improvisation, Scali explains the amount of practice required to trust the music:

As dancers we study technique. We have a whole bunch of basic steps and variations to our steps that help us connect to a partner and to the music. But at a certain moment we just have to allow ourselves to really fall into the music and for the music to take over our bodies. We need to trust, and this is why we train so hard. It is so that we can get to a point where we can feel rhythm in every part of our body. (Personal communication, March 5, 2022)

## Soloing Together

Dancing on a Shine team provides opportunity for salsa dancers to train in technique and expand one’s repertoire for improvisational moments. Learning a shine dance is not simply memorizing choreography for the sake of reproducing it. The aim is to learn detailed principles of salsa motion in various body parts that awaken motile moments of possibility in improvisation. When Ieropoli and Scali approach the task of choreographing a shine dance for their students, their intention is for the music to become visible in each movement. Ieropoli says that

as a choreographer you want to express what the music has to offer as much as possible so that nothing goes unheard or missing. When we see good choreography normally it is because everything in the music is being shown. We can see what we hear. (Personal communication, March 5, 2022)

Without engaging in such bodily training, improvising salsa dancers will pick up the rhythm of a salsa song in a few set patterns and before long, no matter what song is playing, display a certain movement signature, just as we do in our general posture or gait (Foster, 2012). Similarly, Gergen (2021) writes of the limiting power repetitive patterns have on our lives, giving the impression that there is a singularly identifiable self quite separated from others. Yet, the more one opens up to new relational dynamics, in this case experiencing salsa rhythms in soulful connection with musicians, other dancers, and audiences, the more we realize we have the potential to experience many social selves. We can take on different characters, moods, and motional qualities that open us up to new ways of being with one another. To let go of the predicable movement patterns is what somatic educator Emilie Conrad, founder of the Continuum Movement meditation, described as accessing our deeper, primordial intelligence (Conrad, 2007), and which Aboriginal scholar Tyson Yunkaporta (2020) referred to as an ancestral, haptic knowledge beneath the repetitive patterns of automaticity that govern much of daily life. Ieropoli speaks of encouraging our most authentic selves to shine:

it takes time to be able to feel good with whatever technical aspects you’re bringing to your dance where you feel good in your own skin. . . How do I make it my own? How do I express myself throughout this? You don’t have to be the best or the most technical dancer; you just need to give yourself. And that’s where the connection with the music and everything else really comes together. It is when you give yourself that you feel the dance is the most genuine and pure and appealing. People won’t want to stop watching you because they’re pulled into your soul. (Personal communication, March 5, 2022)

## Conclusion

We are aware of the tensions we have barely touched in this all too brief inquiry into salsa rhythms and soul connections. This is the literately critical reflexivity necessary for understanding the limits of a disciplinary partnered movement practice such as Salsa dancing in addressing larger life matters of alterity and social justice. But then there is a relational reflexivity affording release from one’s cognitive encapsulation and lending appreciation of the affectivities of connecting meaningfully with others. There is the need for those in teaching and caring professionals to heed the calls to action in doing well for others. And then there is the call and response exemplified in a Salsa dance practice that suggests a more improvisational, co-emergent dynamic. Each of these tensions is premised on breaking free of the socially constructed, signatured self for the sake of the expansive, relationally flowing self that emerges in *Salsa Dura.*

Rebecca, the first author of this article, dances with a mixed-gender salsa shine team drawing participants from five different countries. The team members range in age from early 20s to mid-50s, measure in height from well under 5 feet to over 6, and are of slim and muscular build to voluptuous body types. Yet, when dancing, these external differences in ethnicity, age, height, and body size take a backseat as the pulse of *Salsa Dura* rhythms get beneath the skin and reverberate deep within the flesh. To dance in a shine team is to share a deeply felt kinesthetic, affective, and energetic connection with one another in spite of such different appearances, social positionalities, and cultural complexions off the dance floor. This intense rhythmicity is not about cultural appropriation and individual capitulation, but about coming together in our differences to express “new vocabularies of self-fashioning” (Kabir, 2020, p. 8) that work outward to potentially reshape perceptions of, and relations with, others otherwise viewed quite differently.

What we have thus intentionally only touched upon is an essentially tactful practice of *alterity*. Shining in *salsa dura* is a manner of being with others, in the first instance other dancers, but also musicians and audiences. By application to wider life circumstances, this practice of rhythmicity and what dancers call soul connection bespeaks a generalizable manner of being *altered* sensitively by that which is beyond ourselves. Those who appear very different can shine too. We and they can enjoy in the rhythmicity of life a soul connection. As Jean-Louis Chrétien (2004) concluded in his visual, oral, and essentially tactile address of the call and the response dynamics writ large, outer directedness, or transitivity, is a greater truth than inner directedness, or what he called reflexivity. “Sensitivity is transitive, not reflexive” (pp. 119–120). We feel for others through the rhythmicity of moving together, in concert with one another. Feelings of pleasure derive from the spacious joy of shining and “not through a reflexivity of the flesh that would be conjectured as its original source” (p. 120). We move most meaningfully with others always in potentially life-expanding, life-altering ways.

Shining through this study of salsa rhythmicity are the motions and emotions of a *relational reflexivity* that offer moments of deeply felt connection with others. We are not perpetuating the historical and cultural erasures that would cast salsa dancing simply as a motional and emotionally beneficial somatic practice (Eddy, 2002); rather, we are inviting the rhythmicity of salsa *dance* to expand the positionality of a critically reflective *stance* from a reflexively *me* to a relationally expansive *we* consciousness. This rhythmic, relational reflexivity may be just enough to begin gathering what Gergen (2021) referred to in the title of his book as *Resources for a World on Edge*.

## Notes

1. The salsa rhythm of 1, 2, 3 5, 6, 7 is visible in the weight transfers between the feet. Oppositional rolling shoulder and figure-8 hip contours draw out the silent 4 and 8 counts as moments of bodily engagement that distinguish walking from dancing.

## References

[bibr1-10778004231176094] Bergonzoni C. (2022). Learning in difference: Difference as a pedagogical tool. Journal of Dance Education, 22(4), 265–268. 10.1080/15290824.2020.1821036

[bibr2-10778004231176094] Bordas J. (2012). Salsa, soul, and spirit: Leadership for a multicultural age. Berrett-Koehler Publishers.

[bibr3-10778004231176094] Buono A. (2022). Fostering somatic movement literacy with young children. Research in Dance Education, 23(3), 316–336. 10.1080/14647893.2021.1879773

[bibr4-10778004231176094] Cancienne M. B. Snowber C. N. (2003). Writing rhythm: Movement as method. Qualitative Inquiry, 9(2), 237–253. 10.1177/1077800402250956

[bibr5-10778004231176094] Chrétien J.-L. (2004). The call and the response ( Davenport A. A. , Trans.). Fordham University Press.

[bibr6-10778004231176094] Conrad E. (2007). Life on land: The story of continuum. North Atlantic Books.

[bibr7-10778004231176094] Du Bois W. E. B. Marable M . (2015). Souls of black folk. Routledge.

[bibr8-10778004231176094] Eddy M. (2002). Somatic practices and dance: Global influences. Dance Research Journal, 34(2), 46–62. 10.2307/1478459

[bibr9-10778004231176094] Foster M. A. (2012). Somatic patterning: How to improve posture and movement and ease pain. EMS Press.

[bibr10-10778004231176094] Fraleigh S. (2015). Dancing becomes walking. In Fraleigh S. (Ed.), Moving consciously. Somatic transformations through dance, yoga, and touch (pp. 50–71). University of Illinois Press.

[bibr11-10778004231176094] Gergen K. (2021). The relational imperative: Resources for a world on edge. Taos Institute Publications.

[bibr12-10778004231176094] Harman V. (2019). The sexual politics of ballroom dancing. Palgrave Macmillan.

[bibr13-10778004231176094] Henry M . (2008). Material Phenomenology ( Davidson S. , Trans.). Fordham University Press.

[bibr14-10778004231176094] Kabir A. J. (2020). Circum-Atlantic connections and their global kinetoscapes: African-heritage partner dances. Atlantic Studies (Abingdon, England), 17(1), 1–12. 10.1080/14788810.2019.1708159

[bibr15-10778004231176094] Leonard A. E. Bannister N. A. D’Souza N. F. (2022). “(Non)dance and (non)math people”: Challenging binary disciplinary identities in education. Research in Dance Education, 23(4), 451–469. 10.1080/14647893.2020.1853692

[bibr16-10778004231176094] Lloyd R. J. (2015a). From dys/function to flow: Inception, perception and dancing beyond life’s constraints. The Humanistic Psychologist, 43(1), 24–39. 10.1080/08873267.2014.952416.

[bibr17-10778004231176094] Lloyd R. J. (2015b). Learning to let go: Phenomenologically exploring the grip & release in Salsa dance and everyday life. The Journal of Dance, Movement and Spiritualities, 2(2), 119–140. 10.1080/14647893.2020.1853692

[bibr18-10778004231176094] Lloyd R. J. (2017). The feeling of seeing: Factical life in salsa dance. Phenomenology & Practice, 11(1), 58–71. 10.29173/pandpr29338

[bibr19-10778004231176094] Lloyd R. J. (2021). The power of interactive flow in salsa dance: A motion-sensing phenomenological inquiry featuring two-time world champion, Anya Katsevman. Qualitative Research in Sport, Exercise and Health, 13(6), 955–971. 10.1080/2159676X.2020.1820559

[bibr20-10778004231176094] Lloyd R. J. Smith S. (2021). A practical introduction to motion-sensing phenomenology. PHENex Journal/Revue PhénEPS, 11(2), 1–18. https://ojs.acadiau.ca/index.php/phenex/issue/view/2263

[bibr21-10778004231176094] Lloyd R. J. Smith S. (2022a). Leaning into life: A motion-sensing inquiry into becoming interactive for life through partnered practices. Journal of Dance & Somatic Practices, 14(1), 91–108. 10.1386/jdsp_00072_1

[bibr22-10778004231176094] Lloyd R. J. Smith S. (2022b). Becoming interactive for life: Mobilizing relational knowledge for physical educators. Frontiers in Sports & Active Living, 3, 1–11. 10.3389/fspor.2021.769031 PMC8790280 35098118

[bibr23-10778004231176094] McMains J. (2018). Queer tango space: Minority stress, sexual potentiality and gender utopias. TDR: the Drama Review, 62(2), 59–77. 10.1162/DRAM_a_00748

[bibr24-10778004231176094] Nyetap C. Littlemore A. Llyod R. Smith S. (2020). The InterActive for Life (IA4L) Resource: A resource for developing social-emotional skills and movement competencies through physical interactivities. https://function2flow.ca/the-interactive-for-life-project/interactivities/

[bibr25-10778004231176094] Renta P. (2014). The global commercialization of salsa dancing and sabor (Puerto Rico). In Hutchinson S. (Ed.), Salsa world (pp. 117–139). Temple University Press.

[bibr26-10778004231176094] Schupp K. (2020). Performing whiteness on the competition stage: “I dance all styles.” Research in Dance Education, 21(2), 209–224. https://doi-org.proxy.lib.sfu.ca/10.1080/14647893.2020.1798395

[bibr27-10778004231176094] Sheets-Johnstone M. (2011). The primacy of movement: Expanded (2nd ed.). John Benjamins Publishing.

[bibr28-10778004231176094] Smith S. J. (2014). Human-horse partnerships: The discipline of dressage. In Gillett J. Gilbert M. (Eds.), Sport, animals, and society (pp. 35–51). Routledge.

[bibr29-10778004231176094] Smith S. J. (2015a). Dancing with horses: The science and artistry of coenesthetic connection. In Carr N. (Ed.), Domestic animals and leisure (pp. 216–240). Palgrave Macmillan.

[bibr30-10778004231176094] Smith S. J. (2015b). Riding in the skin of the moment: An agogic practice. Phenomenology & Practice, 9(1), 41–54. 10.29173/pandpr253

[bibr31-10778004231176094] Smith S. J. (2018). Vital powers: Cultivating a critter community. Phenomenology & Practice, 12(2), 15–27. https://https://doi.org/10.29173/pandpr29365

[bibr32-10778004231176094] Smith S. J. (2019). Bringing up life in horses. Indo-Pacific Journal of Phenomenology, 18(2), 75–85. 10.1080/20797222.2018.1499266

[bibr33-10778004231176094] Smith S. J. LaRochelle K. (2019). Being with horses as a practice of the self-with-others: A case of getting a FEEL for teaching. In Gunnlaugson O. Sarath E. Bai H. Scott C. (Eds.), The intersubjective turn in contemplative education: Shared approaches for contemplative learning and inquiry across the disciplines (pp. 59–61). SUNY Press.

[bibr34-10778004231176094] Smith S. J. Lloyd R. J. (2022). Improvisational interactivity: Moving beneath the ICE. In Hatt B. E. (Ed.), Crushing ICE: Short on theoretical, long on practical approaches to imagination creativity education (pp. 253–270). Friesen Press.

[bibr35-10778004231176094] Snowber C. (2011). Let the body out: A love letter to the academy from the body. In Malewski E. Jaramillo N. (Eds.), Epistemologies of ignorance in education (pp. 187–198). Information Age Publishing.

[bibr36-10778004231176094] Stern D. N. (1993). The role of feelings for an interpersonal self. In Neisser U. (Ed.), The perceived self: Ecological and interpersonal sources of self knowledge (pp. 205–215). Cambridge University Press.

[bibr37-10778004231176094] Stern D. N. (2010). Forms of vitality: Exploring dynamic experience in psychotherapy, the arts, and development. Oxford University Press.

[bibr38-10778004231176094] Williamson A. Sellers-Young B. (Eds.). (2020). Spiritual herstories: Call of the soul in dance research. Intellect Books.

[bibr39-10778004231176094] Yunkaporta T. (2020). Sand talk: How Indigenous thinking can save the world. HarperCollins.

